# Rays in the Shadows: Batoid Diversity, Occurrence, and Conservation Status in Fiji

**DOI:** 10.3390/biology13020073

**Published:** 2024-01-26

**Authors:** Kerstin Glaus, Luke Gordon, Tom Vierus, Natasha D. Marosi, Helen Sykes

**Affiliations:** 1 School of Agriculture, Geography, Environment, Ocean and Natural Sciences, SAGEONS, The University of the South Pacific, Laucala Campus, Suva, Fiji; 2 Manta Trust, Dorchester DT2 0NT, UK; 3 Independent Researcher, Suva, Fiji; tom@vierus.de; 4 Beqa Adventure Divers, Pacific Harbour, Fiji; natasha@myfijishark.com; 5 Centre for Research in Animal Behaviour, College of Life and Environmental Sciences, University of Exeter, Exeter EX4 4SB, UK; 6 Fiji Shark Lab., Pacific Harbour, Fiji; 7 Marine Ecology Consulting, Lami, Fiji; helen@marineecologyfiji.com

**Keywords:** elasmobranchs, participatory science, environmental DNA, biodiversity, Oceania, conservation, distribution, endangered species

## Abstract

**Simple Summary:**

This study compiled data from various sources, including a literature review, participatory science programs, and environmental DNA to assess the diversity and occurrence of batoids in Fiji’s waters. A total of 19 batoid species spanning seven families were identified: 19 through the literature, 12 from participatory science programs, and six via eDNA metabarcoding. Notably, the first photographic evidence of the bentfin devil ray (*Mobula thurstoni*, Lloyd, 1908) in Fiji is provided. Geographic distribution patterns revealed spotted eagle rays and maskrays as predominant in the Western Division. Responses from in-person interviews suggested the presence of sawfishes. This study emphasized the efficacy of a multifaceted approach to elucidate the diversity and occurrence of this understudied taxon in Fiji. In total, 68.4% of the documented species face an elevated risk of extinction based on the International Union for the Conservation of Nature Red List criteria. Caution is advised in interpreting older literature-based records, particularly for the giant guitarfish, giant stingaree, and the absence of sawfish verification. Nonetheless, this synthesis contributes to establishing a comprehensive baseline for understanding and conserving ray populations in Fiji.

**Abstract:**

Over recent decades, elasmobranchs (sharks, rays, and skates) have been increasingly recognized among the world’s most threatened marine wildlife, leading to heightened scientific attention. However, batoids (rays and skates) are relatively understudied, especially in Large Ocean States of the Pacific. This synthesis compiles insights on batoid diversity and occurrence in Fiji’s waters by integrating a literature review, participatory science programs such as the Great Fiji Shark Count (GFSC) Initiative, Projects Abroad Fiji (PA), Manta Project Fiji (MPF), and iNaturalist, along with environmental DNA. Nineteen batoid species from seven families were identified: 19 species from the literature, 12 from participatory science programs, and six from eDNA analysis. Notably, this study provides the first photographic evidence for the bentfin devil ray (*Mobula thurstoni*, Lloyd, 1908) in Fiji. GFSC data indicated the highest species diversity in the Western Division, with spotted eagle rays (*Aetobatus ocellatus*, Kuhl, 1823) and maskrays (*Neotrygon* sp.) being observed most. In-person interviews conducted by PA provided information on the occurrence of wedgefishes and potentially sawfishes. MPF records and iNaturalist uploads were dominated by reef manta rays (*M. alfredi*, Krefft, 1868), while the pink whipray (*Pateobatis fai*, Jordan and Seale, 1906) yielded the most DNA sequences. Overall, 68.4% of the species face an elevated extinction risk based on the International Union for the Conservation of Nature Red List criteria. Although caution is warranted with older literature-based records for the giant guitarfish (*Glaucostegus typus*, Anonymous [Bennett], 1830), giant stingaree (*Plesiobatis daviesi*, Wallace, 1967), and the lack of sawfish verification, this synthesis highlights the effectiveness of a combined methodological approach in establishing a reference point for the diversity and occurrence of this understudied taxon in Fiji.

## 1. Introduction

Elasmobranchs (sharks, rays, and skates) are a functionally diverse subclass of high ecological, economic, and cultural significance, yet they are among the most threatened taxa in the marine environment [1,2]. Many elasmobranch species have conservative life-histories and are vulnerable to extinction due to overfishing, bycatch, habitat loss, and climate change effects [3,4]. In the past decades, elasmobranch populations significantly declined in various environments, including pelagic zones and coral reefs [5,6]. Approximately 19.9% of known batoid species (hereinafter referred to as rays) are threatened with extinction (International Union for the Conservation of Nature, IUCN Red List Categories Vulnerable, Endangered, and Critically Endangered), and 47.5% are listed as Data Deficient [1], with Rhinidae (wedgefishes) and Glaucostegidae (giant guitarfishes) having emerged as the most imperiled marine fish families globally [7]. Although rays constitute the most speciose subgroup of cartilaginous fishes, currently encompassing 26 families with at least 633 valid species [8], and despite the growing scientific interest in elasmobranchs, there is a publication bias towards sharks, leaving rays less understood [9,10]. Numerous data gaps persist, encompassing unresolved taxonomy [9], life-histories, molecular genetics, as well as essential abundance and distribution information [11,12,13,14].

In Large Ocean States of the Pacific, elasmobranchs are culturally relevant [15], and recent explorations in the biodiverse South-West Pacific and Coral Triangle regions have uncovered new elasmobranch species, extended distribution ranges, and species’ re-discoveries [16,17]. In Fiji, over the past two decades, around 30 peer-reviewed papers have covered aspects of shark biology, ecology, and cultural relevance, addressing movement patterns, marine tourism, essential habitats, small-scale fisheries, pollution, trophic ecology, and behavior (see [18,19,20,21,22,23,24,25,26]). Contrastingly, research on rays is at an early stage. Indeed, only a few peer-reviewed publications focusing on reef manta ray (*Mobula alfredi*, Krefft, 1868) tourism and oceanic manta ray (*M. birostris*, Walbaum, 1792) occurrence, and a description of new deep-water skates in the region, are available to date [27,28,29]. Therefore, assessing ray fauna in Large Ocean States of the Pacific, like Fiji, is vital for understanding marine biodiversity and sustaining cultural heritage in Oceania.

One way to gather data on species diversity and occurrence involves participatory science programs, which include citizen science, crowdsourcing, and co-generated science [30,31]. Particularly, marine tourism explores diverse coastal and marine ecosystems, leading to encounters with numerous species [32,33,34,35]. The Great Fiji Shark Count (GFSC) initiative is a notable example, having fostered trust-building, education, outreach, awareness, and promoting best practices for ecotourism [31]. Similarly, the volunteer program Projects Abroad (PA) in Fiji contributed to the identification of essential habitats for juvenile scalloped hammerhead sharks (*Sphyrna lewini*, Griffith and Smith, 1834) in the Rewa Estuary [36], and juvenile bull sharks (*Carcharhinus leucas*, Müller and Henle, 1839) in the Navua and Rewa Rivers [37,38]. Furthermore, Manta Project Fiji (MPF), an affiliated non-profit organization of the Manta Trust, has been gathering distribution and identification data on manta and devil rays since 2012. MPF relies extensively on participatory science, utilizing photographs and videos submitted by divers, snorkelers, and other recreational ocean users. Additionally, citizen science online platforms such as iNaturalist can provide valuable insights into species occurrence [39,40]. However, participatory science programs come with certain drawbacks, including substantial resource requirements, selectivity, dependence on taxonomic expertise, and concerns about underestimating species presence. To overcome some of these challenges, environmental DNA (eDNA) has emerged as a non-invasive method to detect and identify even rare and elusive species in various ecosystems, including marine waters [41]. By amplifying and sequencing DNA fragments naturally released into the environment, eDNA analysis enables species identification through metabarcoding. Thus, eDNA methods have been increasingly applied in recent years and can complement traditional census survey methods [42] and participatory science programs.

Here, we provide the first synthesis of ray species diversity and occurrence in Fiji. The synthesis incorporates a literature review, data compilation from four participatory science programs, and the application of eDNA methods across multiple sites within the Fiji archipelago. Considering recent taxonomic re-classifications of various ray species, this study adopts the current taxonomic nomenclature employed by the IUCN and ‘Rays of the World’ [8], while acknowledging revised names. Lastly, the provided data contribute to the mapping of species distribution and establish a reference point for tracking changes in ray diversity and occurrence. This is particularly relevant considering Fiji’s voluntary commitment, announced at the United Nation Ocean Conference in 2017, towards the conservation and management of all shark and ray species, along with their critical habitats within Fijian waters [43].

## 2. Material and Methods

### 2.1. Study Area

**Figure 1:**
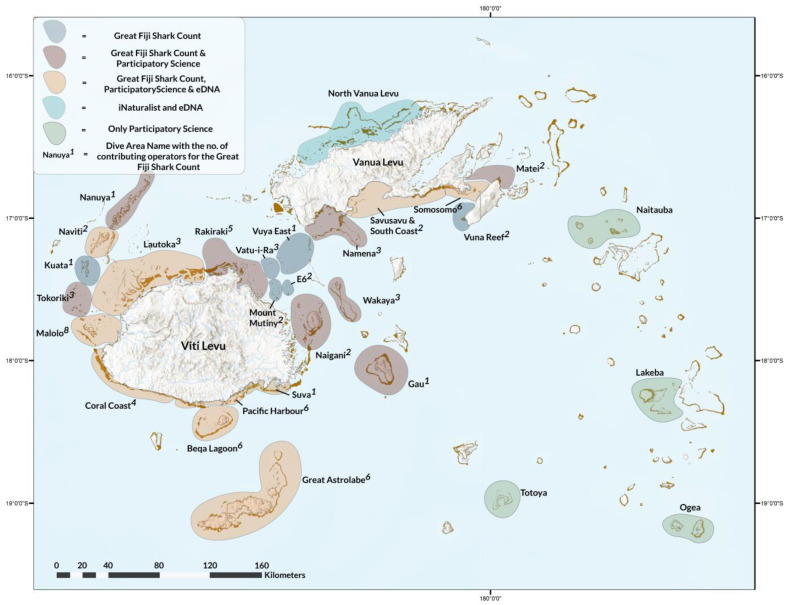
Data collection areas shown, colored by the specific methodologies employed within each geographic area. GFSC is shown separately, and participatory science refers to PA, MFP, and iNaturalist uploads.

The study area was focused on the waters surrounding Fiji located between approximately 12° to 21° South latitude and 176° to 178° East longitude (Figure 1).

### 2.2. Literature Review

Primary data sources encompassed peer-reviewed scientific articles accessed through Google Scholar, from June 2023 to August 2023. The search employed targeted keywords and combinations thereof, namely ‘Rays’, ’Stingrays’, ’Batoids’, ’Sawfishes’, ’Coastal Fisheries’, ’Pelagic Fisheries’, ’Manta Rays’, ’Fiji’, ’Elasmobranch’, ’Fish’, ’Sharks’, and ’South Pacific’. Further information was derived from the gray literature, including but not limited to the Fiji Fishery Resource Profiles [44], a checklist of the fishes of Fiji and a bibliography of Fijian fish [45], a technical report on coastal fisheries in Fiji [46], an in-progress book chapter titled ’Qio kei na vai-sharks and rays of Fiji’ [47], and the guide ’Rays of the World’ [8].

### 2.3. Participatory Science Programs

#### 2.3.1. GFSC

From 2012 to 2019, 39 dive operators across Fiji conducted the first nationwide longitudinal visual census of dive sites for sharks, rays, and turtles, as part of the GFSC in collaboration with eOceans (eOceans.org) [31]. Each April and November, guests (scuba divers and snorkelers) and staff from participating dive operators recorded the details of every dive at 592 sites in 24 areas across Fiji (Figure 1) into community logbooks. Before each dive, dive guides provided guests with instructions about the marine region, the objectives of the GFSC, and presented a field guide to accurately identify the rays that could potentially be encountered in Fiji. Data on the presence or absence of rays at the species level were recorded, whenever possible. For each dive, including all replicates (i.e., multiple people’s observations at the same site simultaneously), additional details recorded encompassed the date, dive times, operator name, site name, maximum depth, and whether spearfishing or wildlife direct feeding was permitted (data not shown here). To assess potential statistically significant variations in ray diversity across the surveyed divisions during the GFSC, a non-parametric Kruskal–Wallis test in R was performed due to the unmet assumptions for a one-way ANOVA [48].

#### 2.3.2. PA

PA is a global voluntourism organization which has been operating international volunteering, internships, and projects for over 30 years. In 2014, PA launched the Shark Conservation Project (SCP), establishing its homebase in Pacific Harbour, Viti Levu, Fiji (Figure 1). PA has worked for over a decade in collaboration with Beqa Adventure Divers (BAD), a shark conservation and research project operating a commercial dive shop in Pacific Harbour to facilitate the ongoing surveys and data collection pursuant to the SCP framework (Supplementary, Figure S1). One pillar of the SCP aims to document the occurrence and abundance of sharks, rays, turtles, and commercially valuable fish through survey dives conducted in the Beqa Lagoon and Pacific Harbour areas (Figure 1). Foundational training was given by PA staff and marine scientists to the volunteers, which included the administration of identification cards containing names (scientific/local), images, and descriptions of all the subject species. As a segway to collecting data on survey dives, PA volunteers needed to successfully complete an exam to ensure they could accurately identify and describe each species. From 2018 to 2020, survey dives were conducted twice a week on Sundays and Wednesdays in the morning and afternoon (weather/conditions and volunteer numbers permitting). For each survey dive, the volunteers were divided into buddy teams of two to three divers per team. The teams utilized dive slates and T-bar measuring sticks to record observed ray species and to estimate their lengths. Numbers of observed ray species were transferred into a Microsoft Excel sheet. Other data collected but not shown here include the following: dive dates, dive durations, site names, regions, maximum depth, species sex (if known), estimated sizes, and occurrence of juveniles of the subject species. A second pillar of the SCP aims to understand the occurrence and abundance of the Critically Endangered bottlenose wedgefish (*Rhynchobatus australiae*, Whitley, 1939) along Viti Levu’s South Coast using a semi-directive interview-based approach [49,50]. In-person interviews were conducted in 2018 and until 2019 with local fishers in Draunikula, province of Serua, led and accompanied by Fijian marine scientists who were staff members at PA. Upon arrival in each village, permission to interview participants was requested from the respective headman and/or the head of clans (Turaga-ni-Koro, Turaga-ni-Yavusa). Headmen and/or head of clans were presented with a sevusevu (kava) as per traditional protocol [51]. Headmen and/or head of clans then designated participants that could be interviewed. Participants were further informed about the main purpose of the survey, that the interview could take up to 30 min, and were assured that all information would be kept confidential and only data aggregates would be revealed for the purpose of the study. Participants were interviewed at their convenience [52], typically in the village meeting halls or their homes. English is an official language in Fiji and interviews were conducted in English and Fijian Bauan, a common dialect which is used throughout Fiji [53]. Information was gathered by means of semi-directive interviews [54]. Pre-categorized and open-ended questions were outlined in a questionnaire (Supplementary, Figure S2). The questionnaire covered various topics including: (i) the socio demographics of respondents; (ii) spatio-temporal information regarding sightings of wedgefishes, including different size-classes; and (iii) potential sightings of guitarfishes and sawfishes. Also, photos of various wedgefishes, giant guitarfishes, and sawfishes were shown to interview participants. Responses were noted by hand on the questionnaires as they were encountered, and no leading questions or a priori coding dictionary were used. Interview responses were transferred into a Microsoft Excel sheet and counted.

#### 2.3.3. MPF

MPF has collected dedicated sighting data, citizen science contributions, and opportunistic sighting data on manta and devil rays across the country since 2012. MPF’s aim is to assess the population dynamics, population demographics, and movement ecology of these rays in Fiji. Sightings are only recorded and added to the national database if either photographic or video evidence is available with clearly visible species-specific identification characteristics as outlined in ‘Guide to the Manta and Devil Rays of the World’ [55]. Sighting data were collected and collated from 23 different sites across Fiji (Figure 1), with six of these sampling sites specifically chosen due to the occurrence of seasonal aggregations of reef and oceanic manta rays [28,29]. An additional 14 sites are the result of opportunistic sightings and citizen science contributions.

#### 2.3.4. iNaturalist

iNaturalist (www.inaturalist.com, accessed on 1 September 2023) is a citizen science platform that was launched in 2008 and became a joint initiative of the California Academy of Science and National Geographic in 2014 and 2017, respectively. Anyone can register and upload photographs of plant or animal species, which are validated through other users confirming or suggesting species identification. Here, the platform was used to examine all ‘research-grade’ batoid species records in Fijian waters up until August 2023, validated by the community.

### 2.4. Environmental DNA Collection and Sample Processing

Considering the critical endangerment of most wedgefishes and the limited knowledge of their status in Oceania [56], sampling sites were primarily selected based on anecdotal or photographic evidence of the presence of these species. From February 2022 to March 2023, 28 eDNA samples (filters) were collected at 17 sites (one replicate sample was gathered at ten sites, with one site sampled three times following on-site wedgefish sightings) spread across Viti Levu, Vanua Levu, Kadavu, Taveuni, and the Yasawa Islands (Figure 1). Twelve samples were collected in the Western Division, 6 each in the Central and Northern Divisions, and 4 samples in Fiji’s Eastern Division. The sampling method consisted of a 2 km-long sampling transect of 30 l to 40 l (30 cm to 100 cm depth), performed during rising and falling tides (one sample each). An ATHENA peristaltic pump and single-use tubing were used to pump the water into a single-use filtration capsule (VigiDNA, 0.2 μm; SPYGEN, Le Bourget-du-Lac, France, www.spygen.com, accessed on 26 July 2023). For each sampling campaign, a strict contamination control protocol was followed in both field and laboratory stages [41]. At the end of filtration, each capsule was emptied, filled with 80 mL CL1 conservation buffer (SPYGEN), and stored in the dark at room temperature before further processing. The DNA extraction was performed in a laboratory at SPYGEN, equipped with positive air pressure, UV treatment, and frequent air renewal. Decontamination procedures were conducted before and after all manipulations. Twelve PCR DNA amplifications were performed per sample. Detailed protocols of DNA extraction, amplification, and sequencing can be found in [41,57]. A teleost-specific 12S mitochondrial rRNA primer pair (Teleo, forward primer—ACACCGCCCGTCACTCT, reverse primer—CTTCCGGTACACTTACCATG) was used for the amplification of metabarcode sequences. The Teleo marker has been shown to be the most appropriate for fish, owing to its high interspecific variability and its short size, allowing the reliable detection of rare and degraded DNA [58,59,60,61]. The obtained sequences were cross-referenced with the existing shark and ray species in GenBank [62,63] as provided by SPYGEN.

Lastly, the conservation status of each species was compiled by adding the IUCN Red List assessment, as well as listings for species appearing in Convention on International Trade in Endangered Species of Wild Fauna and Flora (CITES) and Convention on the Conservation of Migratory Species of Wild Animals (CMS) appendices, along with Fiji’s Endangered and Protected Species Act (EPS) (Table 1).

## 3. Results

### 3.1. Literature Review

Two peer-reviewed papers focused on oceanic and reef manta rays [28,29], one paper described two deep-water species of skates in Fiji [27], one source reported range extensions and records of devil rays for the Indo-West Pacific [64], while another documented ray bycatch in Fiji’s pelagic longline fishery [65]. Overall, limited peer-reviewed scientific data exist on Fiji’s rays, with much of the available information derived from sources such as IUCN assessments [66], ‘Rays of the World’, and the gray literature [45,47,67,68,69,70,71,72,73,74]. A total of 19 different species were identified, excluding sawfishes [75]. Fiji’s coastal and pelagic waters feature whiptail stingrays (Dasyatidae, *n* = 8) as the region’s predominant and largest family, alongside documented presences of pelagic eagle rays (Aetobatidae, *n* = 1), manta and devil rays (Mobulidae, *n* = 5), wedgefishes (Rhinidae, *n* = 1), giant stingaree (Plesiobatidae, *n* = 1), giant guitarfishes (Glaucostegidae, *n* = 1), and softnose skates (Arhynchobatidae, *n* = 2) (Table 1). The pelagic stingray (*Pteroplatytrygon violacea*, Bonaparte, 1832), giant stingaree (*Plesiobatis daviesi*, Wallace 1967), Fijian velvet skate (*Notoraja fijiensis*, Séret and Last, 2012) and longlobe velvet skate (*N. longiventralis*, Séret and Last, 2012) are considered Least Concern, while seven species (~36.8%) are Vulnerable, four (~21%) are Endangered, and two (~10.5%) Critically Endangered. Hence, ~68.4% of Fiji’s ray fauna has an increased risk of extinction. The Oceania fantail ray (*Taeniura lessoni*, Last, White and Naylor, 2016) is Data Deficient, while the species identification for Fiji’s maskray (*Neotrygon* sp.) is pending (personal communication, KG). Various species are included in the CITES and CMS conventions, as outlined in Table 1. These listings serve as additional proxy indicators of conservation concern. Seven species are listed in CITES appendices, and six species are found in CMS appendices, again excluding sawfishes. Except for the giant guitarfish (*Glaucostegus typus*, Anonymous [Bennett], 1830), the same species are listed under both conventions. Fiji’s EPS Act applies to CITES-listed and endemic (indigenous) rays, such as the Fiji velvet skate and possibly the Fiji maskray.

It is further noted in [45] that the common stingray (as *Dasyatis pastinaca*, Linnaeus, 1758) was wrongly listed and that the red stingray (as *D. akajei*, Müller and Henle 1841) was misidentified. The broad cowtail ray (*Pastinachus ater*, Macleay, 1883) was photographed twice as by-catch in longline operations in January and February 2008 [47]. Reports of the green sawfish (*Pristis zijsron*, Bleeker, 1851) in Fiji relied primarily on specimens held in the ichthyology collections of Australian Museums, but the origins of these specimens are questionable, and the species record was deleted for Fiji [75]. However, Fiji’s EPS explicitly lists the largetooth sawfish (as *P. microdon*, Linnaeus, 1758) [63]. Furthermore, the former Chief Scientific Officer at the Fiji Department of Fisheries has reported anecdotal sawfish sightings near Ovalau and between Leleuvia and Caqalai Islands, northeast of Suva (personal communication, HS).

### 3.2. Participatory Science

#### 3.2.1. GFSC

**Figure 2:**
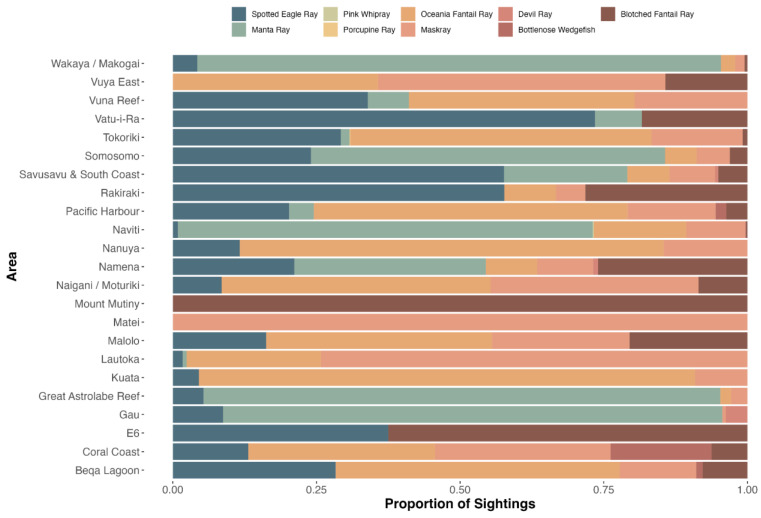
Ray species observations by area during the GFSC, including proportions of sightings.

**Figure 3:**
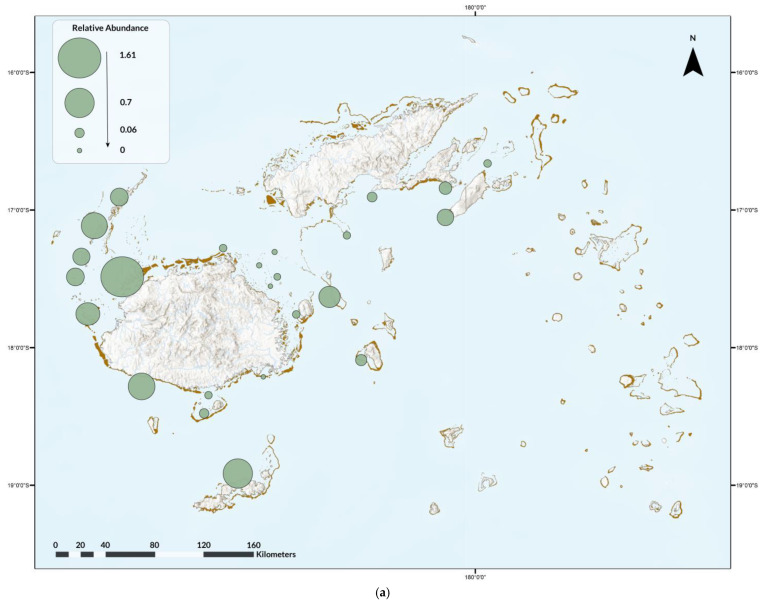

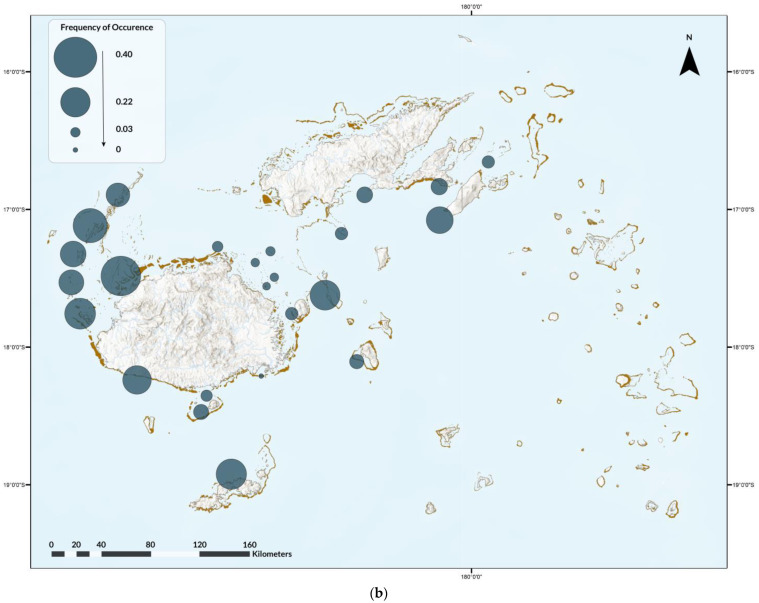
(**a**) Relative abundance of ray species observed during the Great Fiji Shark Count from 2012 to 2019 and (**b**) the overall frequency of occurrence for any/all ray species on dives in that area.

Nine ray species were recorded on 3838 dives (13%), for a total of 2965 rays. Diversity was highest in Naviti, with seven species, followed by Savusavu and South Coast and Pacific Harbour, with six species each, and Tokoriki, Coral Coast, Namena, Somosomo, and Wakaya, with five species each (Figure 2). No ray species were observed in all areas, but, from most to least common by area were the spotted eagle ray (as *Aetobatus narinari*; Euphrasen, 1790, 20 areas) and maskray (as bluespotted stingray, *D. kuhlii*; Müller and Henle, 1841, 20 areas), Oceania fantail ray (as *T. lymma*; Forsskål, 1775, 18 areas), blotched fantail ray (*Taeniurops meyeni*, Müller and Henle, 1841, 16 areas), manta ray (both species, 10 areas), devil rays (three areas), and bottlenose wedgefish (as wedgefish, three areas) (Figure 2). In addition, two pink whiprays (*Pateobatis fai*, Jordan and Seale, 1906) were reported from Naviti in the Yasawas in November 2014 and one porcupine ray (*Urogymnus asperrimus*, Bloch and Schneider, 1801) was reported from Tokoriki in April 2014. The relative abundance and frequency of occurrence varied. Rays were most abundant in the Ba Province (Figure 3a), and most frequently observed in the Western Division and least in the areas of Suva, Vatu-i-Ra, Vuya East, and Mount Mutiny (Figure 3b). The Kruskal-Wallis test revealed no significance between divisions, with *p* = 3.622 × 10^−8^ (Supplementary S4).

**Figure 4:**
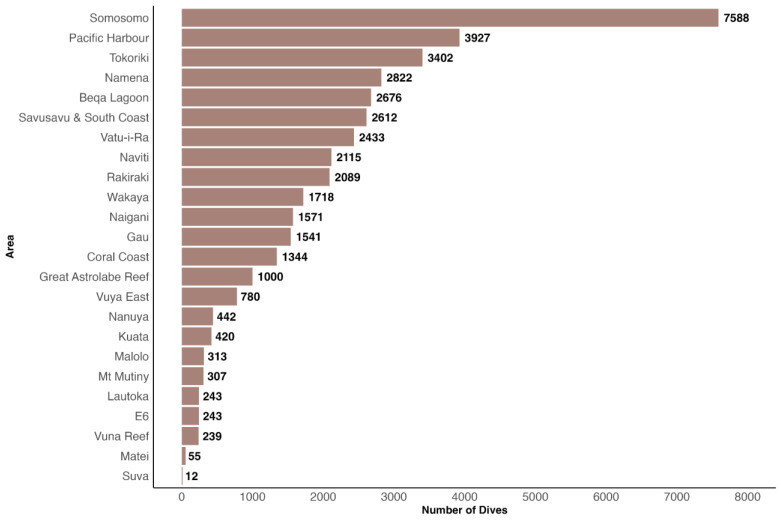
Number of dives performed in each of the 24 surveyed areas during the study period. In total, 39,892 dives were performed.

Survey efforts in terms of dive frequency during the entire study period were most concentrated in Somosomo in the Northern Division, followed by Pacific Harbour in the Central Division and Tokoriki in the Western Division. Conversely, the fewest dives were conducted in Fiji’s capital Suva (Figure 4).

#### 3.2.2. Projects Abroad

Survey dives: PA conducted 272 survey dives in the Beqa Lagoon and Pacific Harbour areas. Rays were observed on 47 of those dives (17%), for a total of 94 rays. Observed rays belonged to either one or a combination of the following three species: Oceania fantail ray (as *T. lymma*), maskray (as *N. kuhlii*, Müller and Henle, 1841), and spotted eagle ray (as *A. narinari*). Observations were as follows: 13 Oceania fantail rays were observed on 13 dives, 75 maskrays were observed on 29 dives, and six spotted eagle rays were observed on four dives. Maskrays comprised the highest number of individuals observed on a single survey dive (*n* = 7).

In-person Interviews: Of the 19 respondents, 10 fishers (52.6%) sighted wedgefishes off Draunikula, including the waters around Yanuca Island. Furthermore, from the interviewed fishers, four (21%) individuals reported sightings of sawfishes both off Draunikula and in their native regions, such as the Yasawa Islands. Based on responses, the observed wedgefishes exhibited varying coloration, including shades of reddish, brownish, or blackish with distinct spots. Reported sightings predominantly occurred during diving activities or as captures in fishing nets. Wedgefishes were reportedly primarily observed lying motionless on sandy bottom areas within reef environments or near the shoreline. Conversely, sawfishes were reportedly more frequently observed in deeper waters at an approximate depth of 30 m, though sightings remain unverified.

#### 3.2.3. Manta Project Fiji

**Figure 5:**
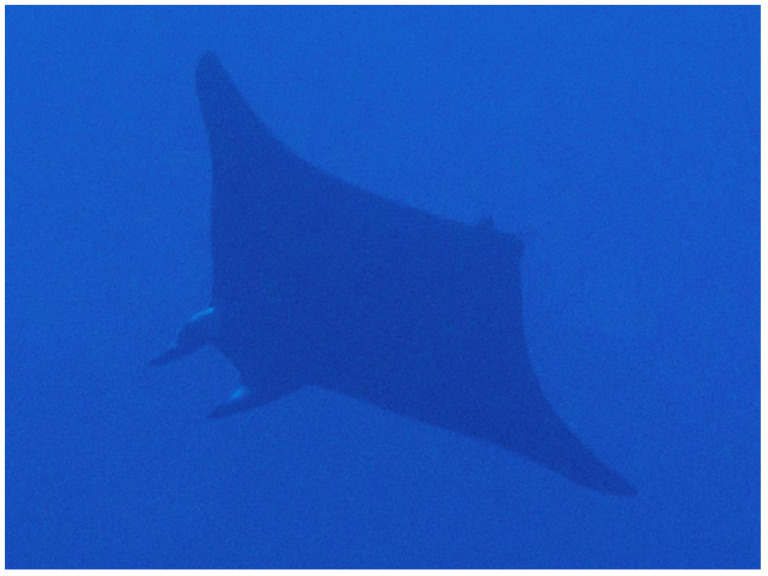
First photographic evidence of the bentfin devil ray in Fiji. Individual displays distinctive double curvature on the anterior margins of pectoral fins, which is a unique identification feature for the species.

A total of 8214 confirmed manta and devil ray sightings of five different species (reef manta ray, oceanic manta ray, sicklefin devil ray, *M. tarapacana*, Philippi, 1892, bentfin devil ray, *M. thurstoni*, Lloyd, 1908, and spinetail devil ray, *M. mobular*, Bonnaterre, 1788) have been recorded since 2012 at 20 different sites across the country (Figure 1). Reef manta rays make up most of these sightings (*n* = 7860). Reef manta rays were also recorded at 21 of the total 23 sites where sightings have occurred. Four of the five manta and devil rays currently identified in Fiji’s waters are listed as Endangered (Table 1). Of these four species, the devil rays are only known from very few observations. For example, the bentfin devil ray is only reported once to MPF, from a sighting in the Namena Marine Reserve near Vanua Levu. Nonetheless, this sighting depicts the first photographic evidence of this species in Fiji (Figure 5). Similarly, the sicklefin devil ray was observed once in the northern Lau Group by a sailor and a spinetail devil ray was sighted on two separate occasions near Drawaqa Island in the Yasawa Islands. The oceanic manta ray is known to occur at Drawaqa Island and Laucala Bay, with the latter identified as a seasonal aggregation site for the species [29]. Additionally, the species has been identified at three more sites: at Malolo Island and Namena Island through opportunistic sightings submitted to MPF; and via tagging data, where a tag was released at Oneata Island in Fiji’s Lau Group from an individual tagged in New Zealand [29].

#### 3.2.4. iNaturalist

**Figure 6:**
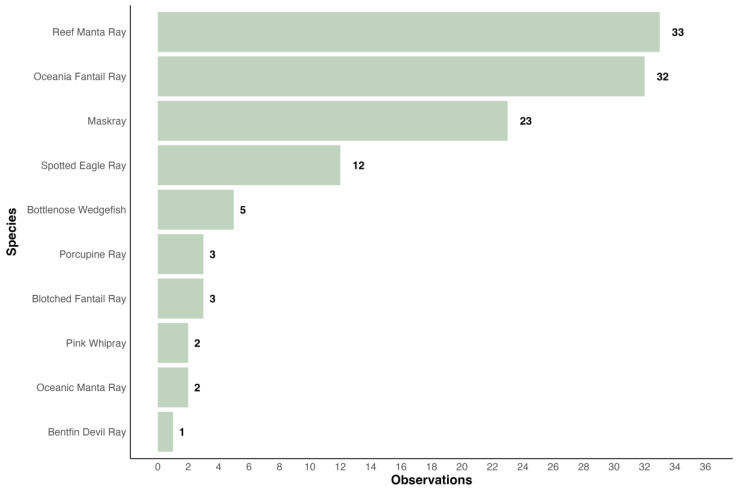
Fiji’s iNaturalist Ray Species Uploads (2006–2023): 124 Observations with 116 ‘Research Grade’ depicted.

A total of 124 observations of ten different ray species have been uploaded to iNaturalist since 2006, of which 116 were validated by other users (Figure 6). All major island groups, except for the Lomaiviti Group, were present (Viti Levu, Vanua Levu, Taveuni, Kadavu, Yasawa Islands, Lau Island Group). Most observations were uploaded for reef manta rays (majority of observations from the Yasawa Islands with several sightings from Wakaya and Kadavu), followed by Oceania fantail rays (observations from several islands with the highest density of sightings in the Mamanuca Islands), maskrays (mostly around Viti Levu but also including Taveuni and Ogea Island), spotted eagle rays, *A. ocellatus*, Kuhl, 1823 (observations from across Fiji), bottlenose wedgefish (observations from Viti Levu, Vanua Levu, and Ogea Island), pink whiprays (observations from Wakaya and Vanua Levu), blotched fantail rays (observations from the Yasawa Islands), oceanic manta rays (observations from Laucala Bay off Suva), porcupine rays (observation from Viti Levu), and a single research grade identification of a bentfin devil ray (observation from Vanua Levu). No records of skates in Fiji were found on iNaturalist.org.

### 3.3. eDNA

**Figure 7:**
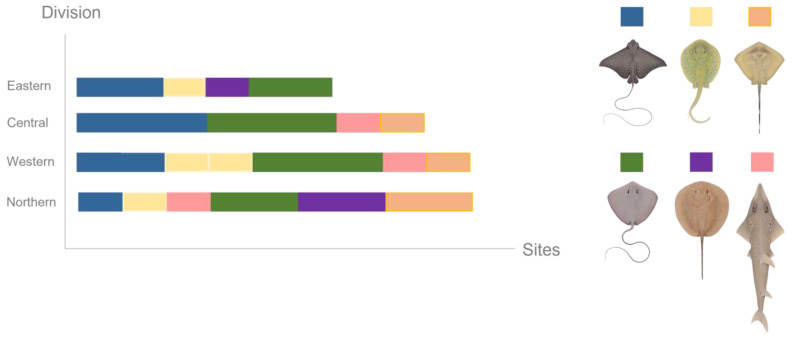
Ray species detected through eDNA methods per number of sites per division.

DNA was collected from six ray species; however, none of the ray species were detected at every site sampled. Despite the lower sampling effort, the Northern Division exhibited the highest ray diversity, with DNA from six ray species, followed by the Western Division, with five identified species. The Central and Eastern Divisions each revealed four species (Figure 7). The pink whipray appeared most frequently, identified in ten (35.71%) out of the 28 samples.

## 4. Discussion

### 4.1. Literature Review

The literature review identifies 19 ray species in Fiji. However, the provided data are coupled with uncertainties. For example, the ‘checklist of the fishes of Fiji and a bibliography of Fijian fish’ includes all and any mentions of species recorded in Fiji and has to be treated with caution [45]. The authors state in their introduction that they were unable to authenticate all records of all the various authors, but express their reservations that the fish may be wrongly identified or is not from this geographical area. Particularly, caution is warranted when considering the credibility of older literature-based records for the giant guitarfish and giant stingaree. The mangrove whipray (*U. granulatus*, Macleay, 1883) is considered extant in Fiji [66], with a patchy but likely wide-ranging distribution in the Indo-West Pacific [8]. However, no further evidence of the in-country occurrence of the mangrove whipray was found. Overall, additional verified reports would enhance the reliability of the giant guitarfish, giant stingaree, and mangrove whipray listings. In a review about sharks and rays in the Solomon Islands, a Large Pacific Ocean State west of Fiji, 18 species of rays were identified [76]. Species found in the Solomon Islands but supposedly absent in Fiji include the coach whipray (*Himantura uarnak*, Gmelin, 1789), sixgill stingray (*Hexatrygon bickelli*, Heemstra and Smith, 1980), dwarf sawfish (*P. clavata*, Garman, 1906), possibly the largetooth sawfish (*P. clavata*, Garman, 1906), and the great torpedo ray (*Tetronarce nobiliana*, Bonaparte, 1865). In Fiji, validated records exist for the bentfin devil ray, spinetail devil ray, Fijian velvet skate, and longlobe velvet skate. Given their range and potential for long-distance migrations, it is likely that the two devil rays also exist in the Solomon Islands. Furthermore, the Solomon Islands, being part of the Coral Triangle, are recognized as a biodiversity hotspot [77]. Fiji is more distant from the Coral Triangle and separated by vast open ocean expanses and deep-water trenches like the New Hebrides trench, which are known barriers to dispersal [78], although some species, such as the great torpedo ray, are capable of extensive pelagic migrations [8]. In conclusion, the absence of scientific data on Fiji’s rays is noteworthy. The authors of this study acknowledge that a substantial portion of the data and observations referred to in this study are derived from their own research efforts. While this may appear self-referenced, it reflects the current scarcity of comprehensive data on rays in Fiji.

### 4.2. Participatory Science Programs

The bentfin devil ray was documented as a new record for Fiji in 2017 [64]; however, where the sightings stem from is not clear. To the best of our knowledge, the image of the bentfin devil ray represents the first photographic evidence of this species in Fiji. The reef manta ray dominated sightings submitted to the MPF, likely due to the ecological needs of the species, resulting in occurrences at easily accessible sites near-shore with a high degree of site fidelity [79] and MPF collecting consistent sighting data at three different species’ aggregation sites across the country. The spotted eagle ray appeared to be widely distributed across Fiji, dominating observations in the GFSC alongside the maskray. Additionally, the spotted eagle ray, maskray, and Oceania fantail ray were prevalent during PA’s survey dives in the Beqa Lagoon. The Oceania fantail ray was commonly observed in the Yasawas, Mamanucas, Viti Levu’s South Coast, Vanua Levu, and in Taveuni. Also, Fiji’s maskray seemed to be prevalent across Fiji, with sightings reported from various islands, including Viti Levu, Vanua Levu, the Lau Group, Taveuni, and Yasawas. The blotched fantail ray, pink whipray, and porcupine ray emerge as elusive species, with infrequent sightings and photographic records. This study did not encompass the examination of potential correlations between environmental variables (e.g., water temperature, depth, salinity, pH) and the occurrence of specific ray species. However, undertaking such correlation analyses is particularly relevant for the identification of ecological drivers influencing ray distributions in Fiji’s waters, and to narrow existing knowledge gaps regarding these understudied species. Altogether, in-country projects, such as GFSC, PA, and MPF, can provide valuable presence/absence data complementing the traditional scientific literature. Additionally, the simplicity and accessibility of international and online-available citizen science projects, such as iNaturalist, further provide insights from a large crowd of nature enthusiasts, naturalists, and other members of society, who previously were not able or willing to share their observations. Moreover, MFP plays a significant part in providing data and advice for management strategies and there are several ongoing community consultations in the Yasawa Islands, Kadavu, and on Viti Levu aimed at creating a set of compulsory interaction rules to facilitate sustainable ecotourism while protecting the resident and transient individuals (personal communication, LG).

#### Species Verification

Overall, species verification and validation through mitochondrial DNA COI barcoding and taxonomic experts are vital to ensure the data’s rigor. The identification of ray species by the MPF and during GFSC and PA dives was considered credible, due to the extensive experience of dive operators, the presence of on-site scientists, and the provision of identification aids in the form of waterproof posters and logbooks. Manta rays have been re-classified with the two traditionally recognized genera, Manta and Mobula, merging after DNA analysis revealed that the traditional classification of Mobula renders it paraphyletic to manta rays [80]. Consequently, manta is now considered a junior synonym of Mobula, resulting in Mobula being the sole extant genus within the Mobulidae family. In-country occurrences of the bottlenose wedgefish [20], Oceania fantail ray, and pink whipray have been confirmed through DNA barcoding (personal communication, KG). The eagle ray has been re-classified as *A. narinari* in the Atlantic, *A. laticeps* in the East Pacific, and *A. ocellatus* in the Indo-Pacific [81,82,83,84]. In a preliminary study, mitochondrial COI barcoding produced over 99% pairwise identity of specimens sampled in Fiji with *A. ocellatus* on the Barcode of Life Data System [85], indicating minor intraspecific genetic variation (personal communication, KG). Despite the variations, the sampled individuals in Fiji are likely to be *A. ocellatus*. However, given previous molecular analyses suggesting potential speciation within the Aetobatus genus, further examination to delineate species boundaries may be needed [86]. The Oceania fantail ray, a recently described and Data Deficient species, further occurs in Papua New Guinea, the Solomon Islands, and in Vanuatu [8,76,87]. Maskrays are native to the Indo-West Pacific region. *Neotrygon* was previously treated as a subgenus of *Dasyatis*. Based on recent morphological and molecular analyses, the subgenus was elevated to the generic level [88] now belonging to the family Dasyatidae. Due to morphological ambiguity, it is often difficult to distinguish between closely related maskrays [89,90], and Fiji’s maskray has traditionally been recognized as Kuhl’s maskray. Ongoing investigations aim to clarify its taxonomic status to provide an accurate species description (personal communication, KG). Over 60% of the species (excluding sawfishes) mentioned in the literature were corroborated by citizen science programs. Discrepancies emerged from the absence of the giant guitarfish, mangrove whipray, pelagic stingray, broad cowtail ray, giant stingaree, and the two skates in citizen science programs. This outcome is plausible considering the deep-water and pelagic habitats of some species (e.g., skates, pelagic stingray, broad cowtail ray, giant stingaree), while records of other species require further validation (giant guitarfish, mangrove whipray, giant stingaree).

### 4.3. eDNA

The DNA of six ray species was successfully captured. However, these detections represent less than a third of the total ray species diversity assumed to inhabit Fiji’s waters. Despite successful detections of many coastal ray species, pelagic species were largely unrecorded, except for the reef-associated semi-pelagic spotted eagle ray. This outcome is largely attributable to the survey focus on coastal waters with reefs, mangrove forests, and seagrass beds. In addition, the variability in DNA persistence in the water column, influenced by factors such as pH, salinity, temperature, UV radiation, microbial activity and others, likely contributed to the limited detection of ray species [91,92]. The transport of eDNA in the environment exhibits significant variability over space and time. Currents, water flow, and turbulence can disperse eDNA unevenly, making it challenging to pinpoint the exact location of a species. As a result, only small segments of genetic material may remain detectable, and the release and decay rates of eDNA can vary widely. Furthermore, the sampling strategy primarily focused on surface waters, which do not adequately represent the habitat preferences of benthic or pelagic rays. Finally, the methodological approach prioritized spatial over temporal coverage. Repeated eDNA sampling at consistent locations and throughout different seasons could potentially enhance the detection and capture of ray DNA traces. Nonetheless, the porcupine ray and the pink whipray were more commonly detected through eDNA methods compared to the GFSC recordings. Also, wedgefish DNA captures reinforce the effectiveness of a combined methodology, as without this direct confirmation, the presence of the species in multiple areas would lack substantiation. Furthermore, eDNA analysis uncovered the highest ray diversity in Fiji’s Northern Division, encompassing areas not surveyed by the GFSC and MPF, providing valuable additional data on ray species diversity. In conclusion, exploring deeper water columns, including benthic and pelagic environments, is recommended for future applications of eDNA methods to assess ray diversity in Fiji’s waters.

Lastly, the validation of methodology also demands careful consideration. The adoption and integration of different methodologies bring distinct advantages to species detection and validation. However, incorporating statistical analyses, along with measures of consistency across the applied methods, would enhance precision in quantifying agreement between different data collection methods.

### 4.4. Wedgefishes and Sawfishes

At least one member of the Rhinidae family has been confirmed in Fiji. The bottlenose wedgefish has been documented in the gray and scientific literature (yet in the former as whitespotted wedgefish, *R. djiddensis*, Forsskål, 1775), observed during the GFSC along Viti Levu’s South Coast, recorded on iNaturalist from Viti Levu, Vanua Levu, and Ogea (Eastern Division), and genetic material was detected in the Central, Western, and Northern Divisions. Together, these findings suggest a likely widespread presence in Fiji. Conversely, there is no confirmation of sawfish presence in the country through photographs, video recordings, or environmental sawfish DNA. Furthermore, verified sightings or captures of sawfishes in Fiji are absent. This reinforces the notion that if sawfishes are present in Fiji, they are rare or occasional vagrants [75]. Still, anecdotal evidence and interview-based collected data suggest the presence of sawfishes. While we acknowledge the deleted species record for the green sawfish as well as the risk of local extirpation [93,94,95], numerous anecdotal accounts of sawfish cannot be discounted. Nevertheless, emphasizing the importance of literature-based, photographic, or genetic evidence, we aim to avoid overinterpreting data sources. Hence, we did not include sawfish in the species count and without verified sightings, sawfishes in Fiji remain elusive.

### 4.5. Conservation

Out of Fiji’s 19 ray species, 68.4% face an elevated risk of extinction. However, the IUCN Red List primarily provides a global conservation perspective, which may not align with regional or local contexts. We acknowledge that the spotted eagle ray is classified as “Least Concern” in the waters of Australia and Oceania, where population density is low, fishing pressure is limited, and specific conservation measures, notably marine reserves, are in place. However, the tendency to include the global assessment instead was based on a number of factors: In Fiji, the species is captured in subsistence and artisanal fisheries (personal communication, KG), population density is not necessarily low, and there are no large marine reserves or other specific conservation measures for this species in place. As highlighted in [76], the conservation status assessments should be considered preliminary, particularly with regard to the unknown population status and trends, lack of clear species knowledge (e.g., maskray), and Data Deficient status (e.g., Oceania fantail ray). Nonetheless, Red List assessments serve as indicators of at-risk ray species in Fiji, particularly when additional data are unavailable. Incidental captures of the broad cowtail ray [47], oceanic manta ray, and pelagic stingray occur in Fiji’s pelagic longline tuna fishery [65]. On a national legislative level, the EPS that Fiji adopted in 2002 regulates the landing and trade of all species as listed on CITES Appendices I, II, and III [68]. Of the species presented in this paper, seven are included in one of the three CITES appendices and thus fall under the jurisdiction of the EPS Act (Table 1). In 2017, the EPS was amended to include non-CITES listed species which are indigenous to Fiji, such as the Fijian velvet skate. If further taxonomic clarifications reveal a distinct species of maskray in Fiji, it would likely be subject to EPS regulations as well. In addition, the ‘Offshore Fisheries Management Act’ (OFMA) adopted in 2012 prohibits the killing, taking, landing, or selling of any species listed in Appendix I and II of CITES [96], which also apply to seven species presented here, making it illegal to catch them (Table 1). While the above-mentioned laws are mostly applicable to offshore fisheries, Fiji’s inshore fisheries are subject to a different set of laws. Similar to other Large Ocean States in the Pacific, Fiji’s coastal fisheries management is primarily carried out at the community level through a marine tenure system, which is based on local authority and self-reliance control [97,98]. The Fiji Fisheries Act (Laws of Fiji, Chapter 158) is applicable to inshore fisheries and grants authority to the Ministry of Fisheries and the Department of Environment for crafting regulations. These regulations can encompass actions like species-specific harvesting bans, demarcation of restricted fishing zones, and oversight of fish stock conservation and protection. The Fisheries Act explicitly prohibits the use of nets, excluding hand nets, wading nets, and cast nets, in estuaries or within 100 m [100 yards] of river/stream mouths. Consequently, enforcement of this existing gillnet ban is vital in safeguarding some of Fiji’s coastal rays, which are caught in small-scale fishery operations (personal communication, KG). Ultimately, a priority remains to ensure the inclusion of rays in coastal fisheries management processes and to advocate for the enforcement of national legislation.

## 5. Conclusions

The synthesis presents an overview of ray diversity, their occurrence and conservation status in Fiji, drawing from the gray and peer-reviewed literature, participatory science programs, and eDNA methods. A total of 19 ray species were identified. To avoid future confusion and to maintain consistency and accuracy, it is strongly recommended to use the updated nomenclature in all future studies and fish lists as outlined in Table 1, including adapting dive logs for participatory science programs, such as the GFSC and PA. Altogether, the careful utilization of available participatory science data is encouraged to paint a more complete picture, especially in data-poor regions. Rather than viewing eDNA analysis as a standalone solution, it should be integrated with traditional survey methods to maximize its benefits while mitigating limitations. Existing ray management measures apply mostly to offshore fisheries, while inshore fisheries are managed at the community level. However, regulatory instruments such as the Fiji Fisheries Act are applicable to these inshore fisheries. This synthesis identifies several key knowledge gaps, which include (1) verification of literature-based records for the giant guitarfish and giant stingaree; (2) verification of sawfishes in Fiji; (3) exploring correlations between environmental variables and ray species occurrence; (4) application of eDNA methods covering benthic and pelagic environments and across different seasons; (5) consistency measures to assess the reliability and consistency of data derived from multiple sources; (6) explicitly considering rays in management arrangements and processes for coastal fisheries; (7) and enhancing compliance and enforcement of existing management, such as the gillnet ban in nearshore areas. Finally, this synthesis can serve as a reference point that can be updated and revised as new data emerge.

## Figures and Tables

**Table 1:** Species of ray reported from the Fiji Islands including a literature review, participatory science methods, and eDNA. The table is organized by Species, Family, IUCN status, CITES, CMS and EPS listings, and previous classifications. Asterisks denote deleted species record for Fiji. Primary and secondary literature sources for each respective species can be found in the Supplementary S3.

Common Name	Family	Scientific Name	IUCN Status	CITES	CMS	Fiji EPS	Methodology Recorded			Previous Classification
				-	-		Literature Review	Participatory Science	eDNA	
Maskray	Dasyatidae	*Neotrygon* sp.	NA			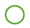	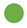	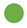	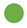	*Dasyatis kuhlii* *Amphotistius kuhlii*
Porcupine ray	Dasyatidae	*Urogymnus asperrimus*	Vulnerable				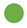	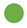	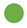	
Mangrove whipray	Dasyatidae	*Urogymnus granulatus*	Vulnerable				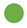			*Himantura granulatus*
Pink whipray	Dasyatidae	*Pateobatis fai*	Vulnerable				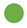	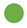	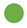	*Himantura fai*
Oceania fantail ray	Dasyatidae	*Taeniura lessoni*	Data Deficient				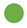	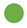	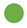	*Taeniura lymma*
Blotched fantail ray	Dasyatidae	*Taeniurops meyeni*	Least Concern				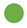	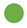		*Taeniura meyeni*
Pelagic stingray	Dasyatidae	*Pteroplatytrygon violacea*	Least Concern				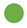			*Dasyatis violacea*
Broad cowtail ray	Dasyatidae	*Pastinachus ater*	Vulnerable				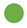			
Spotted eagle ray	Aetobatidae	*Aetobatus ocellatus*	Vulnerable				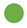	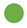	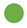	*Aetobatus narinari*
Oceanic manta ray	Mobulidae	*Mobula birostris*	Endangered	Appendix II	Appendices I & II	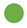	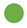	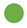		*Manta birostris*
Reef manta ray	Mobulidae	*Mobula alfredi*	Vulnerable	Appendix II	Appendices I & II	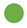	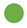	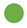		*Manta alfredi*
Sicklefin devil ray	Mobulidae	*Mobula tarapacana*	Endangered	Appendix II	Appendices I & II	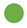	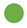	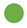		
Bentfin devil ray	Mobulidae	*Mobula thurstoni*	Endangered	Appendix II	Appendices I & II	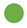	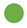	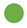		
Spinetail devil ray	Mobulidae	*Mobula mobular*	Endangered	Appendix II	Appendices I & II	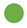	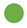	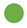		
Fijian velvet skate	Arhynchobatidae	*Notoraja fijiensis*	Least Concern			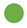	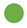			
Longlobe velvet skate	Arhynchobatidae	*Notoraja longiventralis*	Least Concern				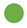			
Bottlenose wedgefish	Rhinidae	*Rhynchobatus australiae*	Critically Endangered	Appendix II	Appendix II	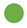	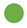	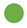	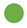	*Rhynchobatus djiddensis*
Giant stingaree	Plesiobatidae	*Plesiobatis daviesi*	Least Concern				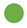			
Giant guitarfish	Glaucostegidae	*Glaucostegus typus*	Critically Endangered	Appendix II		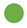	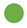			*Rhinobatus batillum*
Sawfishes *	Pristidae	*Pristis* sp.	Critically Endangered	Appendix I	Appendices I & II	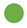	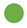	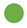		*Pristis zijsron* *Pristis microdon* *Pristis* sp.

## Data Availability

Data indicating specific sites where species occur will not be made available. Other data will be made available upon request.

## Supplementary Materials

The following supporting information can be downloaded at: https://www.mdpi.com/article/10.3390/biology13020073/s1, Figure S1: Project Abroad-Fiji Shark Conservation Management Plan; Figure S2: Project Abroad-Interview Questionnaire; Figure S3: Sources per species; Figure S4: R script.
