# Supplementary material for: Rays in the Shadows: Batoid Diversity, Occurrence, and Conservation Status in Fiji
Source: Biology (Basel). 2024 Jan 26;13(2):73. doi: 10.3390/biology13020073 (PMC10886612; doi:10.3390/biology13020073)
Supplement: Supplementary file 1 [file biology-13-00073-s001.zip › S1_PA-fiji-shark-conservation-management-plan.pdf]

Projects Abroad

Shark Conservation Project

Pacific Harbour - Viti Levu

Fiji

Conservation Management Plan

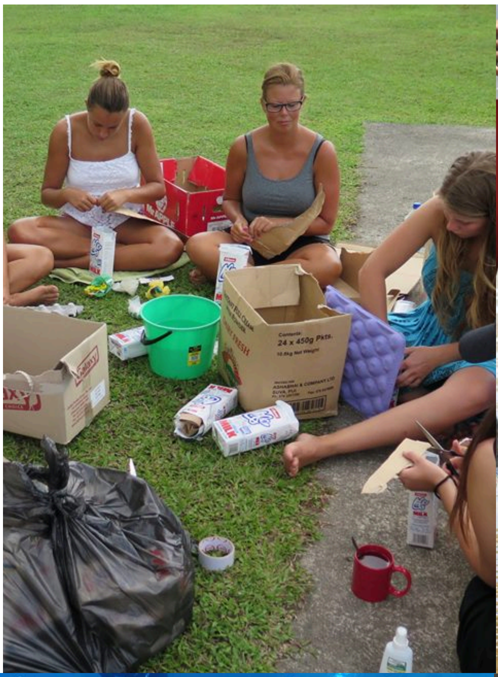

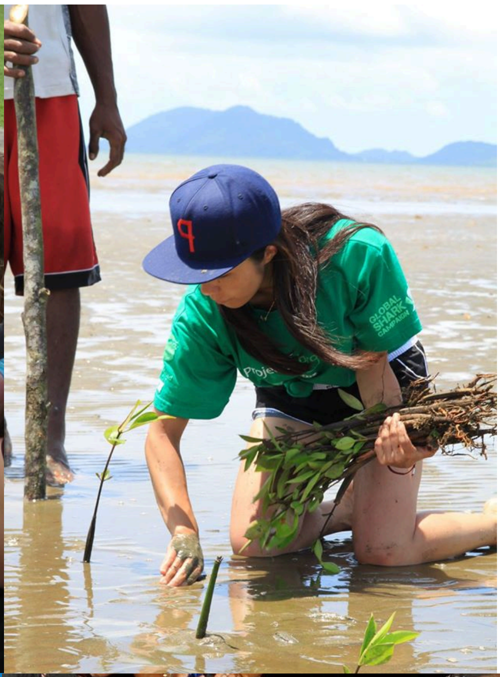

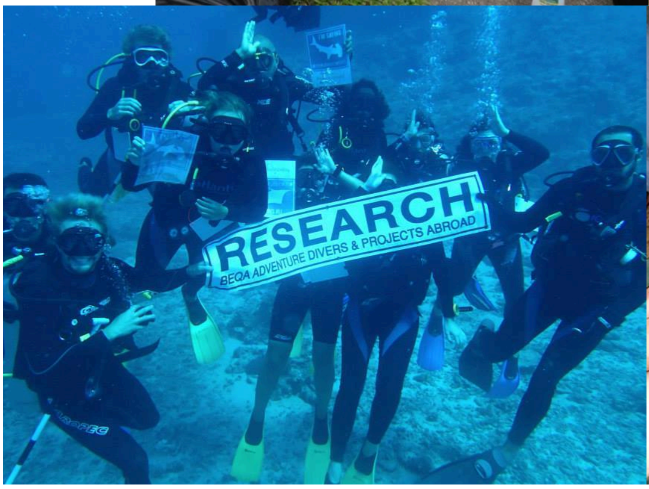

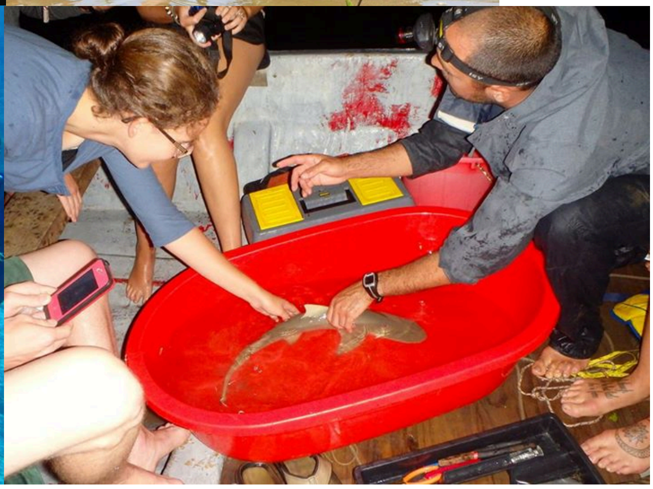

Partners

Content

1 Summary and aim 4

2 Background information 4

3 Working area 5

4 Threats 5

5 Projects 7

5.1 Baited Remote Underwater Video (BRUV) 7

5.2 Survey dives 9

5.3 Shark nurseries assessment 10

5.4 Habitat recovery 11

5.5 Shark identification 12

5.6 Environmental impact mitigation 12

Summary and aim

The Shark Conservation Project (SCP), based in Pacific Harbour, Viti Levu, Fiji, was established in January 2014. Since its start, the SCP has been working together with Beqa Adventure Divers (BAD), the independent researcher Dr. Juerg Brunnschweiler, Dr. Demian Chapman and Dr. Mark Bond from Stony Brook University, and the local communities to carry out scientific research to address shark conservation issues in this area of the South Pacific region. Recently, several other collaborators have joined the effort, such as the World Wide Fund for Nature (WWF) through its Global Shark Program Manager Ian Campbell and the University of the South Pacific (USP) through Professor Ciro Rico. The SCP is also collaborating with the Mangroves for Fiji initiative to replant mangrove seedlings and ensure a healthy and sustainable mangrove forest around Pacific Harbour region. In addition, the SCP has a strong education and awareness program with local communities and schools, about the importance of sharks in maintaining the balance in Ocean's ecosystems. The general aim of the SCP is to generate high quality scientific data to inform local managers and stakeholders about the status of shark populations in the south coast of Viti Levu, and to increase shark awareness among the local communities and general public.

1. Background information

Fiji is an island country about 2000 km northeast of New Zealand, and consists of over 300 islands, of which 110 are permanently inhabited. Because of its topography, the sea is, and has always been, an important source of food and income. In modern days, it has additionally been a growing attraction for tourism, which has in turn lead to an increasingly larger portion of the Fijian economy. Even though local initiatives have already been made to preserve the

wildlife in the ocean, such as the establishment of Shark Reef Marine Reserve (SRMR) outside Beqa Lagoon, overfishing and habitat loss (e.g. coral reef and mangrove forests) are current conservation issues in the country. Mangrove forests are a natural barrier for protecting the coastline against erosion, sea-level changes, storm surges and tsunamis. This key ecosystem also helps to support the adjacent coral reef systems by providing nutrients and, most importantly, being a nursery ground for numerous marine species such as sharks, fish and crustaceans. Therefore, the protection and sustainable use of these vulnerable ecosystems will improve the livelihoods of local communities, protect biodiversity and secure a long-tem ecotourism in the country.

2. Working area

The SCP is based in Pacific Harbour in the south coast of Viti Levu, Fiji. The working area consist of bays, rivers, reefs, and local villages along the coast of Pacific Harbour region, the lagoon of Beqa and its Marine Protected Areas (Red Areas), and Shark Reef Marine Reserve (SRMR) (Fig. 1).

3. Threats

Shark have been roaming the oceans for the past 400 millions years, surviving multiple major extinctions, but in less than a century, we have pushed those creatures to a critical point. “Shark are more vulnerable today than ever before”, said Boris Worm, a professor of biology at Canada's Dalhousie University. An estimated 73 million sharks are killed each year that works out to more than 10 000 sharks per hour. The most significant threats are overfishing, habitat loss and pollution. It is well documented the role of sharks as key species in the ecosystems they inhabit. As apex predators, sharks maintain the balance between the trophic levels of the food chain. Through a cascade effect, a decline in sharks can result an increase in of primary producer

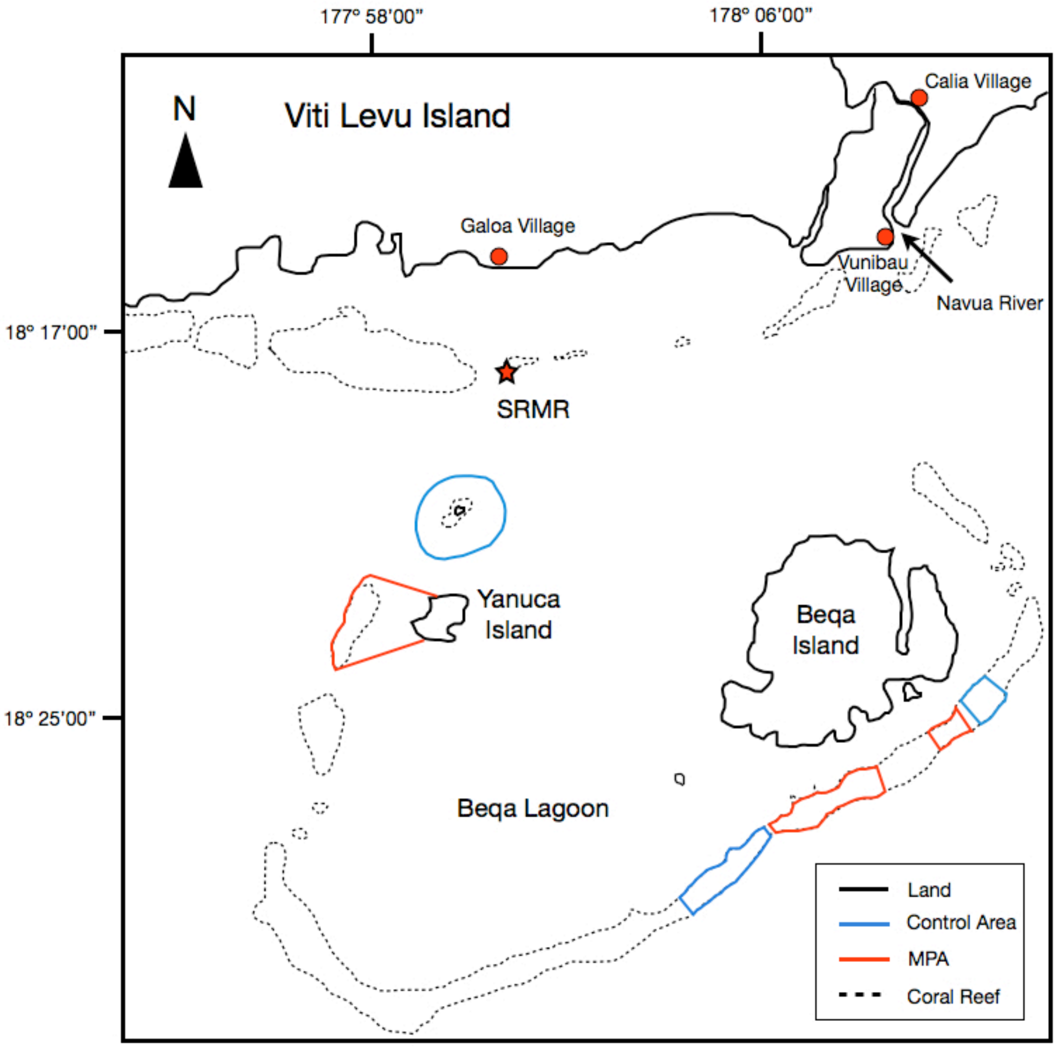

Figure 1: Map of Pacific Harbour region, south Viti Levu, where the SCP activities and research take place.

(algae). This lack of balance has been shown to have negative effects on reefs, and ultimately on the livelihood of local communities. Mangrove forests provide an important nursery ground for commercially valuable species of bony fish and crustaceans, but also for Elasmobranchs (sharks and rays), such as bull sharks (*Carcharhinus leucas*), hammerhead sharks (*Sphyrna spp*.), zebra sharks (*Stegostoma fasciatum*) and guitarfish (*Rhynchobatus spp.*) among others. Despite its value in ecosystem services, mangroves in Fiji are being deforested for its timber, as firewood and building materials, or to make way for urban development, aquacultures and agricultures.

4. Projects

5.1. Baited Remote Underwater Video (BRUV)

Due to the difficulties of studying elasmobranch abundance in the wild, different techniques had to be invented in order to address different ecological questions. One of these techniques is the Baited Remote Underwater Video (BRUV). This shark study method has been used in numerous studies around the world and has been shown very successful.

5.1.1 Objectives

The BRUV project is part of Dr. Mark Bond's research to estimate and compare the relative abundance of predatory fish species, including sharks and rays, inside and outside marine protected areas (MPAs) of the lagoon. These species are also the most commercially valuable and play an important role in the local villages everyday life. Several questions are waiting for an answer through this study: Is there a correlation between diversity and relative abundance of elasmobranchs, and the size of the MPA? Are the MPAs created by NGOs and local villages being successful?

5.1.2 Methodology

BRUVs consist of a video camera (GoPro Hero 3) inside an underwater housing that is mounted on an aluminium frame (Fig. 2). Bait is placed in a wire cage mounted on a pole in the camera's field of view. 1 kg of sardine is used as standardized bait for every single BRUV drop. BRUV sampling is conducted in three different MPAs in Beqa Lagoon and their respective Control Areas (Fig. 1). Locations of deployment for each site are chosen using a random number generator to avoid location bias. BRUVs are placed on the random locations at daylight hours and, upon arrival, the vessel captain is asked to find the closest suitable location for deployment.

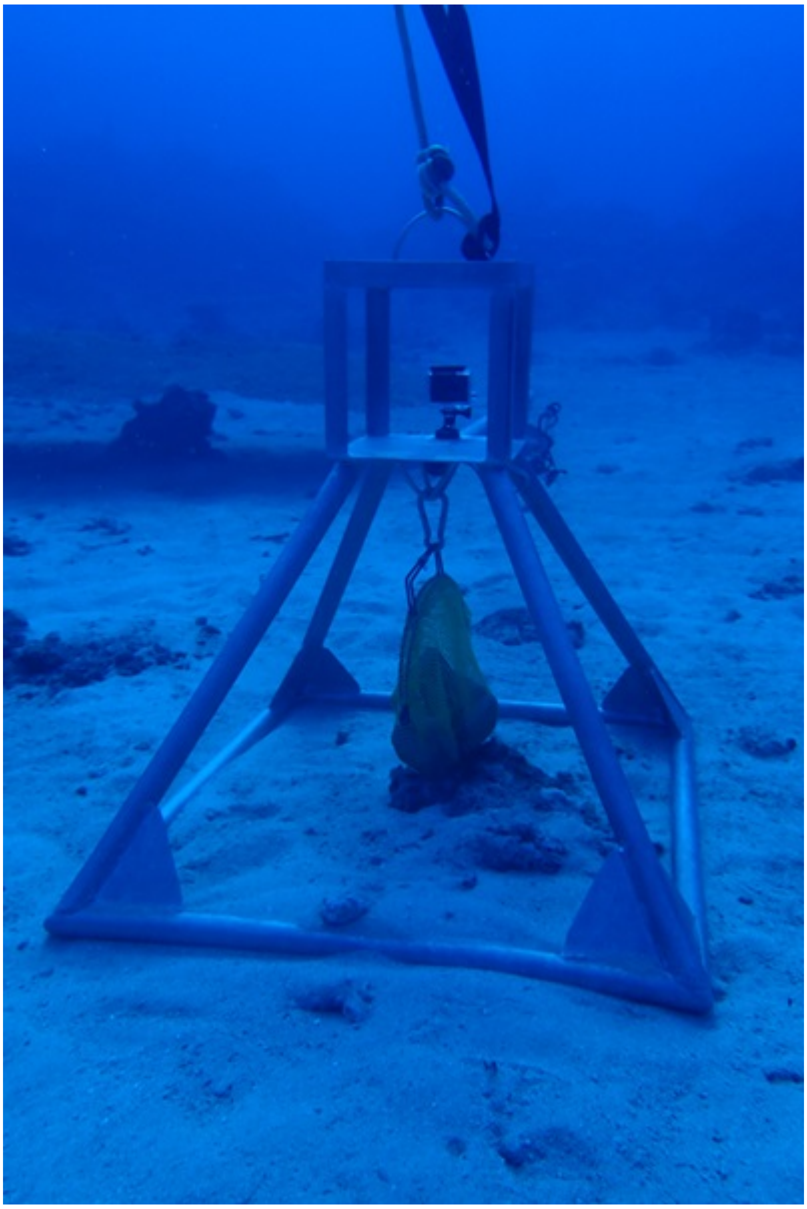

Figure 2: Baited Remote Underwater Video design and drop position facing the direction of the current.

The deployments are typically done in an area between 4 and 30 m of depth, with a flat bottom to maximize line of sight, and at least 10 m away from an edge to prevent from loosing the BRUV due to strong current. BRUVs are deployed using SCUBA gear and diving lift bags to guide it away from live coral and to orient the BRUV facing down current. The BRUV is in the water for over 90 minutes and other activities (e.g. survey dives) are simultaneously conducted within 1 km from the BRUV drop site. At both the start and end of each deployment environmental variables are

measured including, water current speed (with a General Oceanics, Mechanical Flowmeter), bottom depth, underwater visibility and water temperature. BRUVs are dropped 4 times a week until a minimum of 30 drops per study area is reached. Each video is viewed twice by two different pairs of volunteers on a computer and data are recorded. Every elasmobranch or commercially valuable fish species (Appendix I and II) present in the frame is counted and recorded.

5.2. Survey dives

Roaming Survey Dives is a very common technique to estimate abundance of organisms in different ecosystems such as coral reefs. Recently, studies have shown the potential use of survey dives to collect data and when compared to other techniques such as BRUVs or fishing surveys, it showed similar results of abundance of elasmobranchs.

5.2.1 Objectives

The main objective of the Survey Dive Project is to estimate the abundance of elasmobranchs, turtles and commercially valuable fish species (Appendix I and II) in different areas around Beqa Lagoon (Fig. 1). This survey method is complementing the data collected through our BRUV Project.

5.2.2 Methodology

Two survey dives are conducted after every BRUV drop for a total of eight survey dives every week. To do so, the volunteers are divided in two groups: one in training and one collecting data. Volunteers are split into buddy teams of two to three divers inside each group. The data group record all the species of Appendix I and II sightings; estimate their length and take note of their sex if possible. The abiotic variables such as current,

temperature, and visibility are also recorded. Volunteers of the training group are learning diving skills and species that have to be recorded (Appendix I and II). The survey is starting after reaching 5 meters depth and continues for 30 minutes, between 5 and 20 meters depth. After each dive, the data are entered and closely reviewed by Projects Abroad scientists to avoid biases and overestimation of numbers.

5.3. Shark nurseries assessment

Despite the growing interest in the importance of sharks to marine ecosystems, surprisingly little is known about their life habits. Information on breeding and populations is crucial to the creation of management and protection plans for these threatened ocean animals.

5.3.1 Objectives

In collaboration with the University of the South Pacific, Projects Abroad is running a shark-tagging program. The main objective of this project is to find the potential shark nurseries around Viti Levu and assess their quality. Projects Abroad is concentrating its effort around Pacific Harbour region.

5.3.2 Methodology

Collection and tagging of sharks during this study are performed accordingly to regulations provided by the Serua Provincial Council. The tagging team is comprised of at least 1 staff member per boat and volunteers from Projects Abroad. Tagging trips are performed twice a week and last for 5 to 8 hours. Sharks are collected using standardized gears: a 4-inch mesh gill-net and two long-lines (55 m and 75 m) with baited circle hooks. Caught sharks are brought on board, and kept in a tub filled with water. Sharks are measured (pre caudal, fork and total length), sexed and a tissue samples are collected for future genetic analyses. In

addition captured sharks are tagged with a Personal Integrated Transponder tags (PIT Tags).

5.4. Habitat recovery

Mangrove forests are the coastal tropical rainforest where ocean, river and land meet. It's one of the most productive ecosystems on earth, and acts as nursery and habitat for numerous species of fish, crustaceans, mammals and birds. It's also an important carbon sink that counteracts the climate change. Many commercial fish species and elasmobranchs use the mangrove as nursery ground for their juveniles.

5.4.1 Objectives

Projects Abroad aim is to raise awareness and teach about the importance of mangrove forests and about the ecosystem services that provides a pristine forest. These benefits are not always obvious, which makes it even more important to spread the word. We are also be focusing on restoring lost mangrove habitats. By collecting and planting new mangrove seeds in Pacific Harbour region, and soon around Fiji by sending away the propagules, we offset the projects carbon emissions thus helping the project to become more carbon neutral.

5.4.2 Methodology

Volunteers are collecting mangrove propagules from the local area and bring them back to one of our four plant nurseries. The propagules are plant into recycled plastic bottle until they start to develop a root system. This early stage is the most critical period in a mangrove tree's life, and by growing them under controlled conditions we can increase their chance of survival. After 2 to 3 months of germination, volunteers are replanting them in preselected areas. To raise awareness, volunteers are working

closely with the local villages and arrange seminars at hotels and resorts in the area to talk about the importance of the mangrove ecosystem.

5.5. Shark identification

5.5.1 Objectives

The objective of this project is to create a presence/absence database of all the different identified individuals at Shark Reef Marine Reserve (Fig. 1). This type of data allows the volunteers and Projects Abroad team to determine abundance throughout the year and the different cycles and behaviour trend for each individual (e.g. mating scars, pregnancy, site fidelity to SRMR). In addition, this project allows the volunteers to create a bond with sharks that they cannot experience anywhere in the world.

5.5.2 Methodology

Volunteers attend a Shark ID workshop where they learn the identified individuals of SRMR, in particular bull sharks. After training, volunteers perform one Shark Dive at SRMR every month where they count the number of sharks of each species, identify all individuals present at the dive, collect data from different behaviour, and record special markings on each individual shark. Highly trained staff members from Beqa Adventure Divers and Projects Abroad review the data.

5.6. Environmental impact mitigation

Despite being mostly consumed as a single use items, such as cups, food wrappings and bottles, plastic is a material designed to last forever. Right now there is an estimated 100 million tons of garbage in the world's oceans.

5.6.1 Objectives

In conjunction with the Habitat Recovery Project, Projects Abroad is also

conducting a Recycling Project. The objective is to reduce locally the oceanic plastic pollution. This project has been instrumental in involving the local villagers to take responsibility for there own environment. Currently the initiative has collected more than 50,000 plastic bottles, which are repurposed as planting containers for mangrove propagules (seedlings). Over the last few months, a marked reduction in plastic from the villages and surrounding areas has been seen and the effort continues in 2015. Villagers have been seen going through their rubbish, digging and looking for plastic on the beach, forest and even in the surrounding mangroves. The buy back scheme in placed, which involves giving the villagers 3c per bottle, has given an incentive and much needed financial help to the local villages.

5.6.2 Methodology

A crucial aspect of this initiative is the involvement of the local villages and communities in collecting the plastic bottles from the beaches, mangrove forests and their surrounding areas. Projects Abroad volunteers go into a local village to collect and count the plastic bottles. The villagers are then remunerated for their effort. The plastic bottles are then sorted to determine their company of origin. The plastic bottles that we can't reuse as pots are then properly disposed of at the landfill since Fiji doesn't have any recycling program.

The initiative is expanding with the involvement of the government and a dialogue is engaged with Coca Cola to take responsibility for their discarded bottles.

Conservation Appendix I
Recorded Wildlife at Projects Abroad

Teleostei

Scientific name	Vernacular

1. Family Carangidae – Jacks & Trevallys (5)

Caranx ignobilis	Giant trevally
Carangoides ferdau	Blue trevally
Caranx melampygus	Bluefin trevally
Caranx sexfasciatus	Bigeye trevally
Elagatis bipinnulatus	Rainbow runner

2. Family Lethrinidae – Emperors (4)

Lethrinus olivaceus	Longface emperor
Lethrinus miniatus	Sweetlip emperor
Lethrinus laticaudis	Grass emperor
Monotaxis grandoculis	Humpnose bigeye bream

3. Family Labridae – Wrasses (1)

Cheilinus undulatus	Humphead wrasse

4. Family Serranidae – Groupers (10)

Plectropomus leopardus	Leopard coral grouper
Cephalopholis miniata	Coral grouper
Plectropomus laevis	Blacksaddle coral grouper
Epinephelus malabaricus	Malabar grouper

Epinephelus maculatus	Highfin grouper
Cephalopholis argus	Peacock grouper
Epinephelus cyanopodus	Speckled grouper
Variola louti	Yellow-edged lyretail grouper
Variola albimarginata	White-edged lyretail grouper
Epinephelus polyphekadion	Camouflage grouper

5. Family Lutjanidae – Snappers (6)

Lutjanus bohar	Red snapper
Lutjanus gibbus	Humpback snapper
Macolor niger	Black snapper
Macolor macularis	Midnight snapper
Lutjanus monostigma	Onespot snapper
Aprion virescens	Green jobfish snapper

6. Family Sphyraenidae – Barracudas (4)

Sphyraena barracuda	Great barracuda
Sphyraena qenie	Blackfin barracuda
Sphyraena jello	Pickhandle barracuda
Sphyraena forsteri	Bigeye barracuda

7. Family Scombridae – Mackerels & Tunas (3)

Scomberomorus commerson	Narrow-barred spanish mackerel
Gymnosarda unicolor	Dogtooth tuna
Grammatorcynus bilineatus	Double-lined mackerel

Conservation Appendix II
Recorded Wildlife at Projects Abroad

Elasmobranchs & turtles (20)

Scientific name	Vernacular

1. Family Carcharhinidae – Requiem sharks (7)

Carcharhinus melanopterus	Blacktip reef shark
Triaenodon obesus	Whitetip reef shark
Carcharhinus leucas	Bull shark
Carcharhinus albimarginatus	Silvertip shark
Negaprion acutidens	Sicklefin lemon shark
Carcharhinus amblyrhynchos	Grey reef shark
Galeocerdo cuvier	Tiger shark

2. Family Sphyrnidae – Hammerhead sharks (2)

Sphyrna mokarran	Great hammerhead shark
Sphyrna lewini	Scalloped hammerhead shark

3. Family Ginglymostomatidae – Nurse sharks (1)

Nebrius ferrugineus	Tawny nurse shark

4. Family Stegostomatidae – Zebra shark (1)

Stegostoma fasciatum	Zebra shark

5. Family Dasyatidae – Whiptail stingrays (4)

Taeniura lymma	Bluespotted ribbontail ray

Neotrygon kuhlii	Bluespotted stingray (Kuhl’s stinray)
Taeniura meyeni	Giant reef ray (Marbled stingray)
Himantura fai	Pink whipray (Tahitian whipray)

6. Family Myliobatidae – Eagle & manta rays (2)

Aetobatus narinari	Spotted eagle ray
Manta alfredi	Reef manta ray

7. Family Rhinobatidae – Guitarfish (1)

Rhynchobatus laevis	Shovelnose ray (Smoothnose wedgefish)

8. Family Cheloniidae – Sea turtle (2)

Eretmochelys imbricata	Hawksbill sea turtle
Chelonia mydas	Green sea turtle
