# Supplementary material for: Rays in the Shadows: Batoid Diversity, Occurrence, and Conservation Status in Fiji
Source: Biology (Basel). 2024 Jan 26;13(2):73. doi: 10.3390/biology13020073 (PMC10886612; doi:10.3390/biology13020073)
Supplement: Supplementary file 1 [file biology-13-00073-s001.zip › S2_Questionnaire_Projects Abroad_KG.pdf]

Supplementary, S2: Questionnaire

Occurrence of Wedgefishes, Giant Guitarfishes and Sawfishes along the coastal waters of southern Viti Levu, Fiji

Interviewer name:

Team leader name:

Date:

Interview No./ Date:

Interview No./ Total:

Village name:

Coordinates:

Age of respondent:

Gender of respondent:

Part 1: General Introduction:

1. Did you grow up in this area?
2. Are you a fisher?
3. For how long have you been fishing?
4. How often do you go fishing? (i.e. daily / 5-6 times per week / 4-5 times per week / 2-3 times per week / every fortnight / once a month / occasionally)
5. When do you go fishing? (i.e. daytime / nighttime/ incoming tide / outgoing tide / overnight fishing trips)

Part 2: Fishing location

6. Where do you go fishing? (i.e. river / rivermouth / coastline / coral reefs / beyond coral reefs)
7. What is the name of your *qoliqoli*?

Part 2: Wedgefishes, giant guitarfishes, and sawfishes (show ID pictures)

8. Do you see these types of fishes when you are in or close to the water?
    a. If respondent says “YES”, show all pictures of the respective animals
    b. If respondent says “NO”, ask whether she/he has seen the animals of interests in other parts of Fiji. If the answer remains “NO”, please proceed to Q16.

9. Please point at the wedgefishes, giant guitarfishes or sawfishes you have seen (note all species pointed out)
10. Can you please describe in your own words how these fishes look like? (color, size, shape, any special characteristics)
11. Where do you usually see these fishes? (i.e. rivermouths / close to rivers / shoreline / coral reefs / beyond reefs/ etc.)
12. When do you see these fishes? (i.e. nighttime / daytime / low tide / high tide / wet season / dry season)
13. Have you ever seen pups of these fishes? If yes, where and when?
14. Have you also seen these fishes in the past / when you were younger?
    a. If "YES", did you see them more often before or now?
15. Do you know someone who sees them often or who catches them or who can share stories about these fishes? (If "YES", ask village headman to arrange an interview with the respective person).

Part 4: Fishing and Conservation

16. Do you catch these fishes?
17. If yes, what do you use them for? (i.e. subsistence, sale, sharing within the community)
18. How often do you catch them?
19. How do you catch these fishes? (i.e. gillnet / line / rope / spear or speargun)
20. Do you eat these fishes? (ask this question anyway, even if the person does not catch them her- or himself).
21. Are these fishes important to you? (i.e. food source / culture / income)

This is the end of the interview. Vinaka vakalevu. Is there anything you would like to share or any question you would like to ask?
