# Supplementary figures and images for: Rays in the Shadows: Batoid Diversity, Occurrence, and Conservation Status in Fiji
Source: Biology (Basel). 2024 Jan 26;13(2):73. doi: 10.3390/biology13020073 (PMC10886612; doi:10.3390/biology13020073)

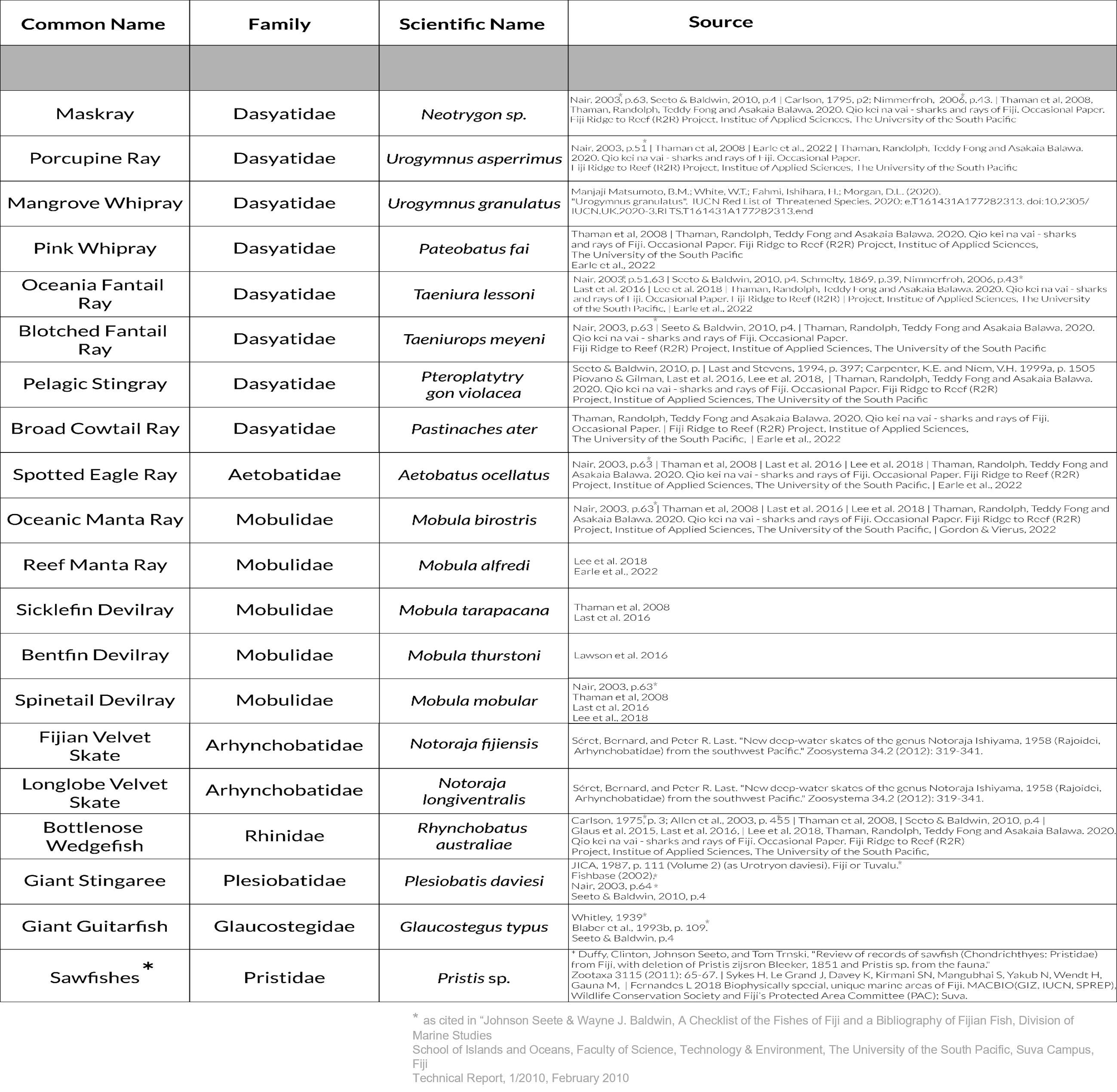

Supplement: Supplementary file 1 [file biology-13-00073-s001.zip › S3_Table2-01.png]
